# Surveillance of Influenza Virus A in Migratory Waterfowl in Northern Europe

**DOI:** 10.3201/eid1303.061130

**Published:** 2007-03

**Authors:** Anders Wallensten, Vincent J. Munster, Neus Latorre-Margalef, Mia Brytting, Johan Elmberg, Ron A.M. Fouchier, Thord Fransson, Paul D. Haemig, Malin Karlsson, Åke Lundkvist, Albert D.M.E. Osterhaus, Martin Stervander, Jonas Waldenström, Björn Olsen

**Affiliations:** * Smedby Health Center, Kalmar, Sweden; † Linköping University, Linköping, Sweden; ‡ Erasmus Medical Center, Rotterdam, the Netherlands; § Kalmar University, Kalmar, Sweden; ¶ Swedish Institute for Infectious Disease Control, Solna, Sweden; # Kristianstad University, Kristianstad, Sweden; ** Swedish Museum of Natural History, Stockholm, Sweden; †† Ottenby Bird Observatory, Degerhamn, Sweden; ‡‡ Lund University, Lund, Sweden; §§ Umeå University, Umeå, Sweden

**Keywords:** influenza A virus, influenza in birds, avian influenza, prevalence, ducks, surveillance, research

## Abstract

We conducted large-scale, systematic sampling of influenza type A virus in migratory waterfowl (mostly mallards [*Anas platyrhynchos*]) at Ottenby Bird Observatory, southeast Sweden. As with previous studies, we found a higher prevalence in fall than spring, and among juveniles compared with adults. However, in contrast to other studies, we found that prevalence in spring was sometimes high (mean 4.0%, highest 9.5%). This finding raises the possibility that ducks are capable of perpetuating influenza A virus of different subtypes and subtype combinations throughout the year and from 1 year to the next. Isolation of the H5 and H7 subtypes was common, which suggests risk for transmission to sensitive domestic animals such as poultry. We argue that wild bird screening can function as a sentinel system, and we give an example of how it could have been used to forecast a remote and deadly outbreak of influenza A in poultry.

## Abstract 2

Ducks may maintain influenza virus from 1 year to the next.

The influenza A virus, including all its subtypes and most of their subtype combinations, is commonly found in aquatic birds such as ducks, geese, gulls, and shorebirds, while only a limited number of subtypes have been found in nonavian hosts. Therefore, waterfowl, in particular wild dabbling ducks (genus *Anas*), are believed to constitute the main natural viral reservoir for low pathogenic influenza A virus, from which strains occasionally arise that are transmitted to other species, including humans and poultry (*1*)

Current knowledge of influenza A virus ecology in wild birds is derived mainly from North American studies (*1*,*2*), which show seasonal changes and between-year fluctuations in prevalence and subtype distribution. Highest incidences occur in juvenile and thus immunologically naïve ducks during fall migration. At other times of the year, however, the observed prevalence is very low, which raises the question as to how the multitude of subtypes are maintained and perpetuated (*1*).

Influenza A virus has diversified into 2 separate avian lineages, North American and European (*3*,*4*), so it is reasonable to ask whether the ecology of influenza A virus in Europe differs from that in North America. Unfortunately, few studies have been conducted in Europe, so more data are urgently needed.

We report results from a 4-year study of influenza A virus occurrence in migrating ducks (mainly mallards [*Anas platyrhynchos*]) in Sweden. We show that prevalence patterns remained similar over the study period but that important differences regarding seasonality and subtype distribution occurred when compared with previous studies from North America. We also compare our data to other long-term systematic surveillance studies of influenza A virus in wild ducks, review geographic patterns and prevalence of influenza A virus subtypes, and discuss their modes of perpetuation in waterfowl.

## Materials and Methods

**Figure 1:**
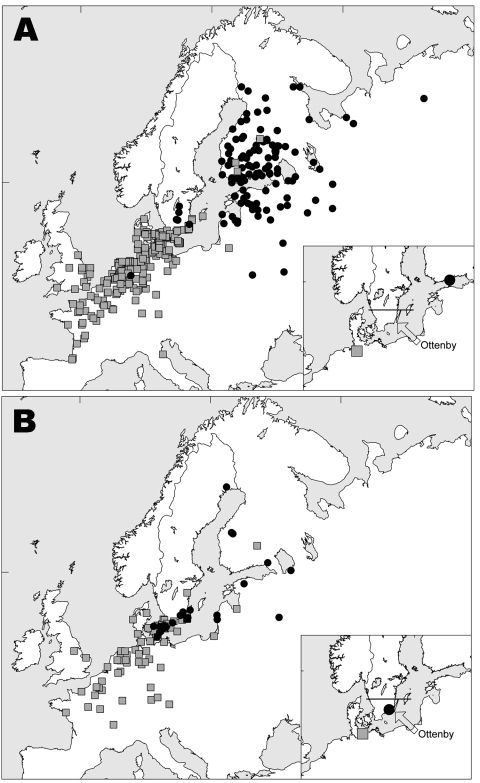
Female mallards banded in Sweden south of 57°30′N (indicated by a solid line in the inserted figures) in Oct–Dec (A) and May–Sep (B) and recovered in winter (Nov–Feb, n = 255 and n = 98) and summer (May–Aug, n = 135 and n = 53). Black dots represent summer recoveries; gray squares represent winter recoveries. Symbols on inset maps represent calculated mean positions and the location of Ottenby Bird Observatory.

From 2002 to 2005, we collected samples from wild waterfowl at Ottenby Bird Observatory (56°12′N, 16°24′E), on Öland, a Swedish island in the Baltic Sea (Figure 1A and B). Birds were caught in a funnel live-trap mainly during migration (see Table 1 for precise dates). We defined the period March–June as spring (comprising the spring migration and some early summering birds), and the period July–December as fall (comprising the fall migration and perhaps some late summering birds). Captured birds were banded with steel rings and identified for species, sex, and (when possible) age. Aged individual birds were assigned to the following categories: fall (juvenile or adult), spring (first spring bird, i.e., juvenile after first winter, or adult) (*5*).

**Table 1:** Sampling data and locations of analyses*

Season	No. mallards screened	Dates	PCR screening location	PCR method used	Subtyping location
2002 Fall	897	9/29–12/16	EMC	Taqman	EMC
2003 Spring	348	4/14–6/30	SMI	Cyber-green	EMC
2003 Fall	755	7/1–12/18	SMI	Cyber-green	EMC
2004 Spring	346	3/26–6/30	KU	Cyber-green	EMC
2004 Fall	789	7.1–12/15	KU	Cyber-green	Not performed
2005 Spring	155	4/3–6/30	KU	Cyber-green	Not performed
2005 Fall	816	7/5–12/13	KU	Cyber-green	Not performed
Total	4,106				

*EMC, Erasmus Medical Center; SMI, Swedish Institute for Disease Prevention and Control; KU, Kalmar University.

### Collection and Preservation of Samples

We placed each captured duck in a box with a clean (unused) paper bottom. Using sterile cotton swabs, we then sampled each bird either by swirling the swab in its cloaca (20% of individual birds) or by swabbing its fresh droppings on the paper bottom. Cotton swabs were immediately put in vials containing virus transport media (Hanks balanced salt solution containing 0.5% lactalbumin, 10% glycerol, 200 U/mL penicillin, 200 μg/mL streptomycin, 100 U/mL polymyxin B sulfate, 250 μg/mL gentamicin, and 50 U/mL nystatin (ICN, Zoetermeer, the Netherlands)) and frozen to –70°C within 30 min.

### Virus Detection

Influenza A virus was detected by 2 different methods (Table 1). Samples collected in fall 2002 were analyzed at the Erasmus Medical Center in Rotterdam, the Netherlands, by using RNA isolation and Taqman as described by Munster et al. (*6*). To ensure efficient influenza A virus detection, the published probe sequence was changed to 5′-6-FAM-TTT-GTG-TTC-ACG-CTC-ACC-GTG-CC-TAMRA-3′, based on avian influenza A virus sequences available from public databases. Pools of 5 individual samples were prepared and processed in parallel with several negative and positive control samples in each run. Upon identification of influenza A virus–positive pools, RNA isolation and Taqman procedures were repeated for the individual samples within each positive pool, and individual Taqman-positive samples were subsequently used for virus isolation.

At the Swedish Institute for Infectious Disease Control (SMI) in Stockholm, we screened samples collected in 2003 by using a real-time PCR (RT-PCR) method directed at the conserved matrix gene with SYBR green technique as developed at SMI (M. Karlsson et al., unpub. data). Some samples from the end of 2003 and samples collected in 2004 and 2005 were screened at the Section for Zoonotic Ecology and Epidemiology, Kalmar University, by using the same method as at SMI with slight local adjustments. The following adjustments were used: RNA was isolated from 100 μL of the original sample by using an EZ1 Virus Mini Kit (Qiagen, Germantown, MD, USA), with the extraction Biorobot EZ1 (Qiagen) set to obtain 75 μL of elution volume. Amplification of the selected part of the influenza A matrix gene was conducted with the LC FastStart DNA Master SYBR Green I kit (Roche Diagnostics GmbH, Roche Applied Science, Mannheim, Germany), having a final reaction volume of 20 L. The thermo cycling was performed in a LightCycler 1.5 (Roche Diagnostics GmbH) under the following conditions: polymerase activation for 10 min at 95°C, and then 43 cycles of 10 s at 95°C, 10 s at 60°C, and 10 s at 72°C. After the amplification, the melting temperature of the PCR product was determined by progressively increasing the temperature from 65°C to 95°C (melting curve analysis).

### Virus Isolation and Characterization

Virus isolation and characterization of positive samples collected in 2002–2004 were performed at the Erasmus Medical Center, Rotterdam. For all influenza A virus RT-PCR–positive samples, 200 μL of the original specimen was injected into the allantoic cavity of 11-day-old embryonated chicken eggs. The allantoic fluid was harvested 2 days after injection, and influenza A virus was detected by using hemagglutination (HA) assays with turkey erythrocytes. When the HA titer was negative, the allantoic fluid was passaged once again in embryonated chicken eggs. Virus isolates were characterized by using a hemagglutination inhibition assay with turkey erythrocytes and subtype-specific hyperimmune rabbit antisera raised against all HA subtypes (*7*).

The neuraminidase (NA) subtypes of influenza A virus isolates were characterized by RT-PCR and sequencing. RT-PCR and sequencing of the NA genes were performed essentially as described by Hoffmann et al. (*8*). Nucleotide and amino acid sequences were aligned by using the Clustal W program running within the Bioedit software package, version 5.0.9 (*9*).

### Mallard Populations and Their Movements

To determine breeding grounds, migration routes, and wintering areas of the mallard populations studied, we analyzed recovery data from all mallards banded (ringed) at Ottenby Bird Observatory from 1962 through 1982 (*10*), and in southern Sweden from 1962 to the present (south of 57°30′N). We obtained these data from the Bird Ringing Centre at the Swedish Museum of Natural History.

Female mallards show stronger philopatry than males, i.e., a higher proportion of the former return to natal areas to breed in consecutive years (*11*). Pair formation takes place in winter, and males that pair-bond with females follow the mate to her natal area. As a consequence, males shift breeding areas between years to a higher degree than females do. We therefore analyzed the banding recovery data according to sex; data on females banded in 1 year and recovered in consecutive breeding seasons were used to outline the general breeding range of mallard populations that pass Ottenby. Recoveries were further divided into birds trapped in summer (May–September) or fall (October–December). All recoveries with uncertainties concerning the date were excluded. The mean geographic position for the different groups was calculated according to Perdeck (*12*). The breeding season was defined as May–August and the winter season as November–February.

## Results

### Sampling Overview

**Figure 2:**
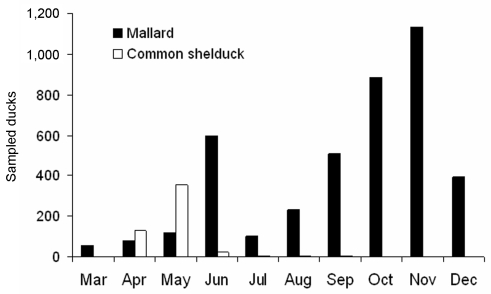
Seasonal variation in the number of sampled mallards (black bars) and common shelducks (open bars). Data from 2002–2005 have been pooled.

We collected samples from 4,800 individual waterfowl of 16 species (Table 2). Only mallard, common shelduck (*Tadorna tadorna*), northern pintail (*Anas acuta*), and Eurasian teal (*A. crecca*) yielded >20 samples each (Table 2). Most (85.5%) of the samples were from mallards. Twice as many birds were sampled in fall (3,323) as in spring (1,477). In fall, 78% of birds aged were juveniles. Most birds were caught during the peak migratory periods of October–December and May-June (Figure 2).

**Table 2:** Number of processed samples and influenza A virus prevalence in waterfowl (*Anatidae*) sampled at Ottenby Bird Observatory, 2002–2005

Species		Spring	Fall	Total	No. positive	Prevalence, %
English name	Scientific name					
Mute swan	*Cygnus olor*		9	9		
Greylag goose	*Anser anser*	2	1	3		
Barnacle goose	*Branta leucopsis*		6	6		
Brent goose	*B. bernicla*		12	12		
Common shelduck	*Tadorna tadorna*	504	7	511	14	2.7
Eurasian wigeon	*Anas penelope*		16	16		
Gadwall	*A. strepera*	1	1	2		
Eurasian teal	*A. crecca*	18	44	62	8	12.9
Mallard	*A. platyrhynchos*	950	3,156	4,106	575	14.0
Northern pintail	*A. acuta*	2	28	30	3	10.0
Common pochard	*Aythya ferina*		2	2		
Tufted duck	*A. fuligula*		18	18		
Common eider	*Somateria mollissima*		15	15		
Long-tailed duck	*Clangula hyemalis*		1	1		
Goldeneye	*Bucephala clangula*		3	3		
Red-breasted merganser	*Mergus serrator*		4	4		
Total		1,477	3,323	4,800	600	

### Mallard Populations and Their Movements

Mallards banded in southern Sweden in the fall belonged to a different population than those banded there in summer. Females caught in the fall months of October–December were usually found east of the Baltic Sea the next breeding season (May–August) in Finland, Russia, and the Baltic States (Figure 1A). In contrast, females banded in summer (May–August) were usually found in nearby areas of Sweden or in Denmark the following breeding season (May–August) (Figure 1B).

Both groups of females wintered mainly in coastal areas of western Europe, from southern Sweden to France and Great Britain; the mean recovery position of females banded during late fall (Figures 1A) was located more to the southwest than that of females banded in summer (Figure 1B). Recoveries of males showed a general pattern similar to that of females but with much more geographic scatter, as predicted from the gender differences in philopatry.

### Prevalence Overview

**Figure 3:**
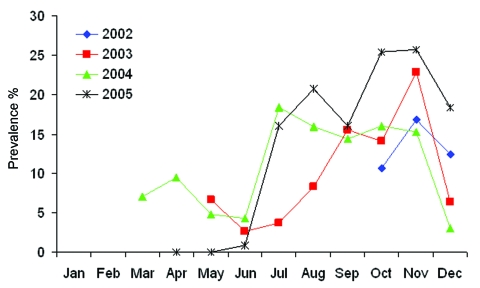
Seasonal influenza A virus prevalence in mallards (n = 4,106) in the 4 study years. Data from months represented by ≤5 samples are not included.

**Figure 4:**
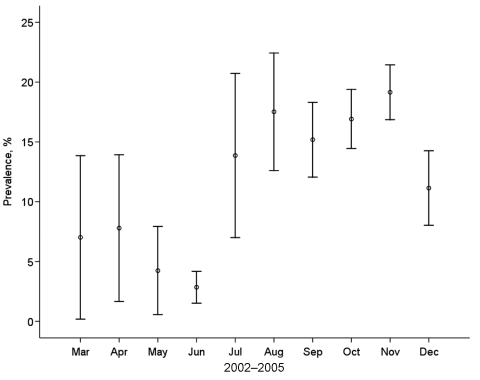
Monthly average influenza A virus prevalence in mallards (n = 4,106), 2002–2005, with bars indicating the standard error. Data from months represented by ≤5 samples are not included.

Total prevalence of influenza A virus in all waterfowl sampled during the 4-year period was 14.5%. However, 575 (95.8%) of the 600 influenza A virus PCR-positive samples were from mallards, and only 25 samples came from other host species (Table 2). Prevalence in mallards at different seasons varied among years but followed the same general pattern, i.e., lower values in spring and early summer compared with late summer and fall (Figures 3 and 4). The highest overall prevalence was found in October 2005 (25.7%) and the lowest in April and May 2005 (0).

### Seasonal Differences

Monthly prevalence in mallards was higher in fall (3.0%–25.7%) than in spring (0–9.5%). Mean fall prevalence (15.0%, n = 2,714) was significantly higher than the corresponding spring value (4.0%, n = 817), both when data were analyzed for each year separately (proportion infected vs. noninfected 2003–2005; χ^2^_1_ = 19.0–41.1, n = 971–1,135, p<0.001) and for the combined dataset of 4 years (χ^2^_1_ = 93.1, n = 4,106, p<0.001).

### Species Differences

Mallards were caught in substantial numbers in spring as well as in fall, whereas common shelducks were caught mainly in spring (Figure 2) and Eurasian teal and northern pintail mainly in fall. Regardless of this caveat, and the much smaller n values, the prevalence rates of these species were similar to those of mallards. For instance, spring prevalence in common shelducks was 2.8%, similar to the 4.0% seen in mallards. Likewise, fall prevalence rates of northern pintails and Eurasian teal, 10.7% and 18.2%, respectively, were within the range of such rates in mallards.

### Age and Sex Patterns

**Figure 5:**
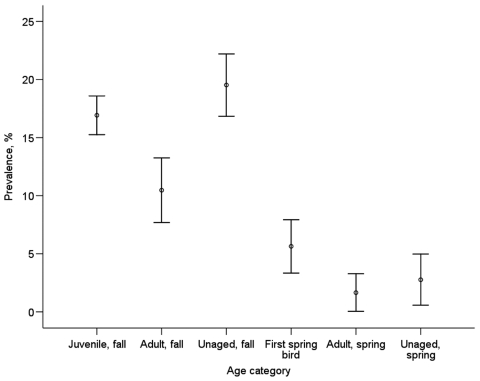
Mean influenza A virus prevalence in the 4 age classes. Birds that we were unable to age correctly were denoted as unaged. Bars indicate standard error.

Looking only at the species with the largest dataset (mallard), we also found differences between age groups. In fall, 11.7% of adults (n = 468) and 20.4% of juveniles (n = 1,944) were positive for influenza A virus. In spring, 1.7% of birds aged as second spring or older (n = 242) and 6.0% of the first spring birds (n = 390) were positive for influenza A virus (Figure 5). Adult birds had consistently lower prevalence than younger birds, both in fall (χ^2^_1_ = 11.41, n = 2,412, p = 0.001) and in spring (χ^2^_1_ = 5.05, n = 632, p = 0.025). We could not detect any differences in influenza A virus prevalence between male and female mallards in either of the 2 comparisons in which the sample sizes permitted statistical testing (juveniles fall: χ^2^_1_ = 3.16, n = 1,944, p = 0.076; adults fall χ^2^_1_ = 0.00, n = 468, p = not significant).

### Subtype Overview

**Figure 6:**
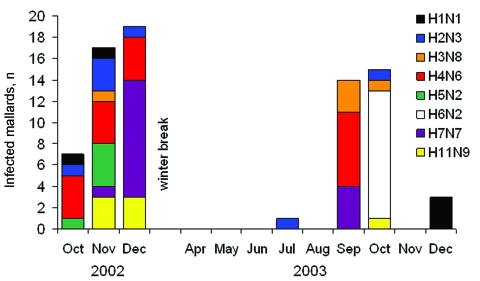
Occurrences of the most common influenza A virus subtype combinations (≥5 isolates) in mallards over time.

Of 213 mallard samples positive by RT-PCR during the first 2 years of sampling, 129 could be isolated by egg culturing. During this period, 11 different HA subtypes and all 9 NA subtypes were found in 40 different subtype combinations. Isolates for 39 of the subtype combinations came from mallards (Table 3). An additional subtype, H3N3, was found in a sample from a Eurasian teal among the 6 isolates obtained from Eurasian teal and common shelducks. All H5 and H7 virus strains were characterized as low pathogenic. The most prevalent combinations were H4N6 (14.7%), H7N7 (12.4%), and H6N2 (9.3%) (Table 3). While most subtype combinations were isolated only during short periods, H4N6 and H2N3 were isolated during longer periods (3 months) (Figure 6).

**Table 3:** Influenza A virus subtype combinations in mallards sampled at Ottenby Bird Observatory, 2002–2004*

	*Neuraminidase*									*Total*
Hemagglutinin	1	2	3	4	5	6	7	8	9	
										
1	5	2				1				8
2	2		7		1					10
3		1				1		5		7
4		3				19				22
5		5	3			1			3	12
6	2	12			1	1		3		19
7							16		1	17
8				4						4
9										0
10		2		1	1	2	2	2	3	13
11	1	1	2		1	1		1	7	14
12					2				1	3
13										0
14										0
15										0
16										0
Total	10	26	12	5	6	26	18	11	15	129

## Discussion

Our large dataset came mainly from mallards, a species known to play a central role in the perpetuation of influenza A virus in nature (*13*). Because the greatest numbers of ducks were caught in fall, juvenile birds predominated in the sample. This finding reflects the age structure of the mallard population at that time, when juvenile birds making their first migration typically outnumber adults. In spring, the ratio of young ducks to older ones is smaller because juveniles experience higher mortality over winter (*14*).

Our banding analysis confirms that mallards migrate from breeding areas in northwestern Russia and Fennoscandia to wintering areas in northwestern Europe. Mallards from breeding areas close to the Baltic Sea start migration to their wintering areas in August–September (Figure 2), while the population in eastern Finland and Russia starts to appear in southern Sweden beginning in October. The wintering area of the latter mallards was, on average, more to the southwest than that of conspecifics breeding in southern Sweden, thus showing a leapfrog migration pattern (Figure 1A and 1B). The distance between breeding and wintering grounds of the northeastern populations is >3× longer than that of the more “resident” population in southern Scandinavia. The chance of receiving reports of banded birds found in Finland has probably been much higher than in Russia because of lower human population density in parts of western Siberia. Thus, a larger proportion of the mallards that pass southern Sweden during October–December might come from breeding areas in western Russia than is actually indicated by the recovery distribution.

Influenza A virus was present in a significant proportion of migrating mallards, in both fall and spring. The prevalence in all mallard samples during the entire 4-year study was 14.0%. The total prevalence of influenza A virus in our study showed consistent patterns across years and seasons (Figures 3 and 4); up to 25.7% of the ducks were infected in fall compared with up to 9.5% in spring.

We compared our results to those of multiyear studies from North America (*2*) and Germany (*15*). However, because so few multiyear studies have been made, all 4 may merely show some of the variation that can be found everywhere, rather than differences between Europe and America. Results from recent studies conducted in other parts of North America support this suggestion (*16**,**17*).

Overall prevalence in our study was similar to that found in the 2 studies mentioned above. However, important differences also occurred. In North America, mallards had the highest prevalence of influenza A virus (10%–60%) in August and September (*1*); this rate dropped sharply in subsequent months to <1% in winter and spring (*18*). In our study, influenza A virus was detected from August to December with peaks in October–November (3.0%–25.7%) and with comparatively high prevalence in most spring months (range 0–9.5%).

Different theories are offered to explain how the various subtypes can be perpetuated in North America, despite a low prevalence in spring. For example, influenza A virus is suggested to survive in frozen lakes and to reinfect birds when they return in spring to breed (*19*). Alternatively, influenza A virus might be carried by other bird species during the months in which the prevalence in ducks is low. In North America, shorebirds in the Delaware Bay area had a 14.2% prevalence of influenza A virus during spring migration and could thus bring influenza A virus back to the ducks’ breeding areas (*2*,*20*).

Our study shows that, contrary to the findings in the North American study (*2*), influenza A virus in migrating dabbling ducks might be perpetuated by the ducks themselves. This view is based on our findings of influenza A virus prevalence of up to 9.5% in some spring months and is also supported by the 8% influenza A virus prevalence at breeding grounds in eastern Siberia (*21*) and a 4.1% influenza A virus prevalence among wintering mallards in Italy (*22*).

In our study, only mallards were caught in substantial numbers in both spring and fall. Thus, our conclusions about seasonal patterns are limited to this species. However, data from the less numerous ducks showed similar frequencies of influenza A virus prevalence.

In mallards, the higher prevalence in juvenile ducks suggests that they are more prone to be infected with influenza A virus. This may reflect their immunologic status and indicates a key role for juveniles in the perpetuation of influenza A virus, as suggested by Hinshaw et al. (*23*). This age difference remained in spring, when adult birds had significantly lower infection rates than second-year birds.

We found all 9 NA types and 11 of 16 recognized subtypes of HA. These were isolated in 40 different combinations (Table 3). Such diversity of influenza A virus subtypes is similar to that found in other large studies of wild duck populations (*2*,*15*). In our study, the HA subtypes H4, H6, and H7 were the most common, followed by H1-H3, H5, H10, and H11. The H8 and H12 subtypes were rarely isolated, and subtypes H9 and H13–16 were never isolated.

The subtype distribution in our study shows both similarities and differences to that found in studies in Germany and North America (*2,**15*). The H4 subtype was common in all 3 studies, but while our study and the North American study share a high number of H6 isolations, the German study had higher numbers of H1 and H2 (Table 4). The fact that some subtypes such as H13 and H16 were not found in our survey or in the other surveys may indicate a difference in host preference for these subtypes, as suggested by other studies (*7,**20*). A high prevalence of influenza A virus of the H7 subtype was found in the German study as well as in ours, but ours had a higher prevalence of the H5 subtype.

**Table 4:** Comparison of multiyear influenza A virus screening studies of ducks in North America (*2*), Germany (*16*) and Sweden (present study)*

Study region	Sweden	Germany	North America
Prevalence during fall	15.0% (in mallards)	8.7%	22.2%
Prevalence during spring	4.0% (in mallards)	No data	0.03%
Most prevalent HA subtypes	H4, H6, H7	H4, H2, H1, H6, H7	H6, H3, H4
HA subtypes not found	H9, H13–16	H5, H12–16	H13–16
Most prevalent NA subtypes	N2, N6, N7	N1, N3, N6	N8, N2, N6
NA subtypes found	N1–9	N1–9	N1–9
Most prevalent subtype combinations	H4N6, H7N7, H6N2	H2N3, H4N6, H1N1, H6N2, H7N7	H3N8, H6N2, H4N6

*HA, hemagglutinin; NA,neuraminidase.

In our study, NA subtypes N2, N6, and N7 dominated, while N4 and N5 were uncommon. As in the German data, N1 and N3 were less prevalent and N2 and N7 more prevalent. In North America, N2, N6, and N8 were the most frequent; N7 was rarely isolated (Table 4).

Although we only present subtype data from 2 complete sampling years, we detected just as many subtype combinations as found in the 12-year German study (*15*). The most prevalent subtype combinations we found, H4N6, H7N7, and H6N2, were also found in the German study. Similarly, both the H4N6 and the H6N2 subtype combinations were among the most common in the North American survey (*2*). The H4N6 subtype stands out as being prevalent in ducks worldwide and across years (Table 4). The H7N7 combination was common in both the German survey and in ours. However, this combination was never isolated in the North American survey, although it has been isolated in another North American study (*16*).

Most subtype combinations we found in fall were short-lived or varied in prevalence over time. Only the H2N3 and H4N6 subtype combinations in the fall of 2002 (Figure 6) showed a constant occurrence. These data, combined with those about mallard migration patterns (Figure 1A and 1B), suggest that different duck populations arriving from different breeding areas may bring different subtype combinations with them. The subtype combinations found late in the fall, for example, were probably brought in by mallards that arrived from breeding areas farther east.

In late fall 2002, we observed a sharp increase in the number of migrating mallards that carried H7N7 (Figure 6). If one assumes that these birds followed their normal migration route after leaving Ottenby (Figure 1A and 1B), they would be on their wintering grounds in western Europe a few months later. At that time, in February 2003, a large H7N7 epizootic in poultry began in the Netherlands. This epizootic also caused human illness, including 1 fatal case (*24*). Retrospectively, we found that the H7N7 samples from our Ottenby ducks were closely related phylogenetically to the H7N7 that caused the outbreak (*6*). Our data suggest that an increase in the incidence of H7 or H5 viruses among wild birds might signal an increased likelihood for transfer to poultry and that bird observatories such as those at Ottenby could play an important role as early warning systems.

Science has barely scratched the surface of the ecologic–virologic–epidemiologic interface of influenza A virus. Further research needs to focus on how the influenza A virus affects individual fitness, vital rates, and population structure in wild ducks, for both low as well as for highly pathogenic strains.
